# Health-related quality of life in paediatric arterial hypertension: a cross-sectional study

**DOI:** 10.1186/s12887-018-1120-0

**Published:** 2018-05-01

**Authors:** Tadej Petek, Tjaša Hertiš, Nataša Marčun Varda

**Affiliations:** 1 Faculty of Medicine, University of Maribor, Taborska ulica 8, 2000 Maribor, Slovenia; 2 Department of Paediatrics, University Medical Centre Maribor, Ljubljanska ulica 5, 2000 Maribor, Slovenia

**Keywords:** Arterial hypertension, Paediatric, Health-related quality of life, PedsQL

## Abstract

**Background:**

The prevalence of paediatric hypertension is increasing worldwide, especially due to the childhood obesity epidemic, and is an important public-health concern. While the Health-Related Quality of Life (HRQoL) was already shown to be impaired in the adult hypertensive population, a scarcity of data still exists on HRQoL in paediatric hypertensive patients. Our purpose was thus to assess the HRQoL of children and adolescents with arterial hypertension, using self- and proxy-reports, and to determine the correlations between child and parent questionnaire scores.

**Methods:**

The Paediatric Quality of Life Inventory™ 4.0 Generic Core Scales were administered via post to children and adolescents, aged 5-18 years, with primary or secondary arterial hypertension and parents as proxy-reports. Patients were recruited from a paediatric nephrology unit in a tertiary hospital, using an out-patient clinic visit registry. Healthy school children and adolescents from a local primary school, aged 6 to 15 years, and their parents formed the control group. HRQoL group comparisons were calculated with independent samples t-test and child-parent correlations with the Pearson’s r correlation coefficient.

**Results:**

In total we recruited 139 patient and 199 control group participants as self- and proxy-reports. Scores from self- as well as proxy-reports indicated a significantly lower overall HRQoL in the paediatric hypertensive population (95% CI for mean score difference: − 11.02, − 2.86 for self- and − 10.28, − 2.67 for proxy-reports; *p* = .001). In self-reports, lower physical (95% CI: -13.95, − 4.89; p = <.001), emotional (95% CI: -12.96, − 2.38; *p* = .005), school (95% CI: -11.30, − 0.42; *p* = .035), and psychosocial functioning scores were observed (95% CI: -10.34, − 1.89; *p* = .005). Parent proxy-reports were lower in physical (95% CI: -14.31, − 5.39; p = <.001), emotional (95% CI: -12.39, − 2.60; *p* = .003) and psychosocial scores (95% CI: -9.36, − 1.34; *p* = .009). Pearson’s r values ranged between 0.62 to 0.79 in patient and 0.56 to 0.80 in control sample (*p* < .001). Interestingly, hypertensive children reported lower social functioning scores than hypertensive adolescents (*p* < .001).

**Conclusions:**

This cross-sectional study gives insight into the detrimental impact of hypertension on children’s and adolescents HRQoL, which may inform public health experts. Furthermore, it shows that clinicians should aim to improve patients’ physical and psychosocial well-being throughout their development.

## Background

Paediatric hypertension is becoming an increasing public-health concern, especially in light of the growing childhood obesity epidemic [1–3]. The prevalence rates of pre-hypertension and hypertension with repeated screenings are rising [4] and are estimated at 9-12% and 2.5–3%, respectively, which makes elevated blood pressure one of the most common paediatric health conditions [1].

It is well-established that children with elevated blood pressure are more likely to grow into hypertensive adults in the future [5]. In addition, it has been shown that target organ damage, including left ventricular hypertrophy and pathologic vascular changes, develops already early on in the disease process [2, 6]. Proper management of high blood pressure in children and adolescents is based on the recommendations of the European Society of Hypertension from 2009 [7], encompassing both lifestyle changes and in cases of symptomatic hypertension, secondary hypertension, hypertensive organ damage, or diabetes, pharmacological therapy.

As measures of Health-Related Quality of Life (HRQoL) have been increasingly developed in the recent years and have to some extent already come to use in paediatric clinical practice [8], several childhood chronic conditions have been shown to negatively influence HRQoL [9]. Whereas there is increasing knowledge regarding clinical aspects of childhood hypertension, a scarcity of data exists on the psychosocial aspects of the disease burden.

In the case of hypertension, paediatric and adult studies report conflicting results, with hypertensive children and adolescents in the “KiGGS” study in Germany [10] scoring higher with self- and parent-rated quality of life measures, as opposed to adults, who report a decrease in HRQoL [11]. Also, in children with mild-to-moderate chronic kidney disease, statistically (but not clinically) significant associations between elevated blood pressure and lower parent and child HRQoL scores were observed at baseline measurement, but did not persist over time [12].

Our aim was therefore to assess the HRQoL of hypertensive children and adolescents in comparison to a normotensive control sample. Based on comparison of several paediatric quality of life instruments [13–15], we have decided on the Slovene translation of the Paediatric Quality of Life Inventory™ (PedsQL) 4.0 [16–18], which is well-established in the paediatric quality of life research and provides good feasibility, reliability and validity of the results. In addition, we aimed to assess child-parent score correlations and finally, compare PedsQL™ scores of healthy children and adolescents to scores from neighbouring countries.

## Methods

### Study design and setting

The study was coordinated by the Nephrology Unit of the Department of Paediatrics, University Medical Centre Maribor, Slovenia. Using a cross-sectional design, we conducted a postal survey of children and adolescents with arterial hypertension and their parents as proxy-reports. The perception of patients’ quality of life was compared to that of a control group, consisting of children and adolescents recruited from a local primary school and their parents. After gaining approval from the hospital Ethics Committee in October 2014 and obtaining permission to use the PedsQL™ 4.0 Questionnaires from Mapi Research Trust in November 2014, we have subsequently procured a list of paediatric hypertensive patient clinic visits from January 2010 to December 2014.

### Patient sample

To be eligible to participate in the study, hypertensive children and adolescents had to be aged between 5 and 18 years at the time of the survey completion, have a diagnosis of primary or secondary arterial hypertension, defined as systolic or diastolic blood pressure above published age-, sex- and height adjusted 95th percentiles, based on measurements from at least 3 occasions [7], and be physically and mentally capable of answering the age-appropriate questionnaires, based on the child’s health condition and parents’ judgement. We did not include the stage of arterial hypertension, neither have we collected data on duration of the disease. Upon meeting eligibility criteria and obtaining oral consent from parents, we sent the families the self- and proxy-versions of the questionnaire, together with the information letter described below, in May 2015. We reached an adequate sample size by the end of the data collection period in December 2015.

We sent all study participants an information letter on the background of the study and the questionnaire administration guidelines, adopted from the PedsQL™ website [19]. The parents were especially advised to let their child fill in the questionnaire separately, without comparing their results. In each case, one parent and their child filled in the questionnaire. Parents of young children were given detailed instructions on administering the questionnaire to their child, in addition to a sheet with a 3-point smiley rating to clarify the meaning of the answers to their child.

### Control sample

The control group consisted of healthy children and adolescents, aged 6 to 15 years, attending a local primary school. Based on reports of health, performed during routine preventive health programmes, we excluded children and adolescents with either a history of arterial hypertension, other chronic diseases or illnesses, and significant acute diseases which could affect one or more questionnaire scores. After gaining consent in November 2015, we have given the children the PedsQL™ questionnaires, together with a sheet with questions about the child’s health in the past month and the before mentioned information letter. After completing the questionnaires, children returned them to the school social worker, who forwarded them to us by the end of December 2015.

### Measures

For the purpose of the study, we used the Slovene translation of the PedsQL™ 4.0 Generic Core scales [19]. It assesses respondents’ physical (8 items), emotional (5 items), social (5 items) and school functioning (5 items) and consists of parallel child self-report and parent proxy-report questionnaires with developmentally-appropriate child categories; ages 5-7 for young children, 8-12 years for children and 13-18 years for adolescents. The PedsQL™ 4.0 Child-Self Report and Parent-Proxy Report Questionnaires are available free of charge for not funded academic research via the ePROVIDE™ Mapi Research Trust website [20].

Asked about the frequency of a specific problem during the past 1 month, respondents could choose the appropriate answer on a 5-point response scale, except with the young child questionnaire, where a 3-point scale was used. Returned surveys were then anonymized and the survey items reverse-scored. Answers were combined into four Scale Scores (Physical Functioning, Emotional Functioning, Social Functioning and School Functioning Scale Score) ranging from 0 to 100, where higher scores indicate a better HRQoL. Two additional Summary Scores were calculated, the Total Scale Score and the Psychosocial Health Summary Score, the latter comprised of the Emotional, Social and School Functioning Scale Scores.

### Outcomes

The primary interest of the study was performance on the Total Scale Score and the Psychosocial Health Summary Score. Secondary outcomes were performance on the Physical Functioning, Emotional Functioning, Social Functioning and School Functioning Scale Scores. Additionally, we calculated child-parent questionnaire score correlations and compared control sample PedsQL™ Scores to those of Slovenia’s bordering countries.

### Bias

We used an internationally accepted questionnaire with good psychometric properties in order to minimize information bias. Response bias was minimized by re-calling the non-responders and resending them the questionnaires. Also, the control group had to fulfil specific criteria regarding their health status, thereby avoiding the assumption of “general well-being” in the school population.

### Study size and statistical methods

To estimate the required number of participants, we have first decided on the “minimally clinically important difference” (MCID) of 5 PedsQL score points, taking into account the previously reported MCID’s [16, 21]. Expecting a total score of approx. 8o points for the control group with an control group/patient group enrolment ratio of 1.5, a significance of *p* = 0.05 and a power of 80% (β = 0.2), the web-based ClinCalc Sample Size Calculator [22] suggested a total sample of 130 participants, with 52 participants in the patient and 78 participants in the control group.

For the statistical analyses, we used the IBM SPSS Statistics software (version 20), running on a personal computer with Windows 10 operating system. According to the PedsQL™ Scoring Algorithm, we would have to discard any questionnaire with more than 50% of the items in the scale missing, but that was not the case in any of the returned questionnaires. We calculated descriptive statistics with means and standard deviations for questionnaire scores and percentages for categorical variables (age, gender, and group). In comparing the means between patient and control groups from self- as also proxy-reports, we used the independent samples *t*-tests (Student’s *t*-test in equal and Welch’s *t*-test in cases of unequal variance). Pearson’s *r* was calculated to estimate child-parent concordances.

## Results

### Sample characteristics

**Fig. 1:**
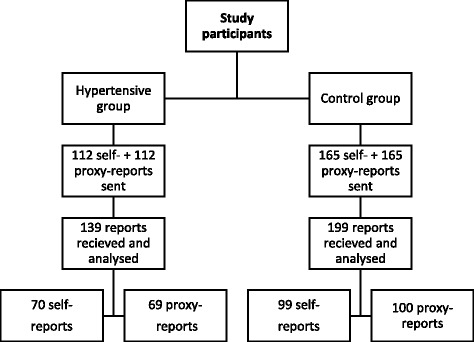
Recruitment of study participants

We have sent a total of 554 questionnaires (see Fig. 1). The hypertensive sample of 112 children and adolescents and their parents was obtained from a list of 510 clinic visits during the 2010-2014 time period. While all of the phone-contacted eligible parents were willing to participate, two children were not eligible due to a recent diagnosis of a brain tumour in one and an intellectual disability in the other case. In the hypertensive sample, a total of 70 children and adolescents (response rate 62.5%) and 69 parents (response rate 61.6%) have answered the questionnaire. From the hypertensive participants, 17 (24.3%) were young children, 18 children (25.7%), and 35 adolescents (50.0%). Most (*n* = 43, 61.4%) were boys, with 10 (58.8%), 10 (58.8%) and 23 (65.7%) boys belonging to young child, child, and adolescent age groups, respectively.

From a total of 330 questionnaires given to children from a local primary school as control samples, we have received 99 self-reports (60.0% response rate) and 100 parent-proxy reports (60.6% response rate). Twelve children were not eligible as controls due to illnesses in the previous month. In control sample self-reports, 27 (27.3%) were young children, 26 (26.3%) children and 46 (46.5%) adolescents. Among them, there were a total of 46 (46.5%) boys, with 14 (51.9%), 10 (38.5%) and 22 (47.8%) boys belonging to young child, child, and adolescent age groups, respectively.

We did not exclude any questionnaires due to missing responses, as all participants had answered more than half of the items in a scale. In calculating Pearson’s *r* correlations, we used 69 child-parent pairs in the hypertensive and 98 pairs in the control sample.

### Outcomes

In comparing PedsQL™ scores of hypertensive children and adolescents to healthy control group using Student’s and Welch’s *t*-tests, several clinically and statistically important differences were found (see Table 1). Most importantly, self-reports in the patient group were 6.94 (SD = 2.06) points lower in the Total Score (95% CI: -11.02 to − 2.86; *p* = .001) and 6.11 (SD = 2.14) points lower in the Psychosocial Health Summary Score (95% CI: -10.32 to − 1.89; *p* = .005). Most affected health domains were the Physical Functioning Scores with a 9.41 score decrease (SD = 2.28; 95% CI: -13.95 to − 4.89; *p* < .001) and the Emotional Functioning scores with a 7.67 score decrease (SD = 2.67; 95% CI: -12.96 to − 2.38; p = .005). School Functioning Scores were lower by 5.86 score points (SD = 2.75; 95% CI: -11.30 to − 0.42; *p* = .035).

**Table 1:** Results of the Student’s and Welch’s t-tests, comparing hypertensive and control group scores

		Patient sample		Control sample		Mean Difference^a^	Effect size	Independent samples *t*-test			
PedsQL 4.0 Generic Core Scales	No. of items	N	Mean (SD)	N	Mean (SD)	Score Points (SED^b^)	Hedges’ *g*	t	df	*p*	95% CI for Mean Difference
Child Self-report											
Total Score	23	70	77.72 (14.57)	99	84.66 (11.00)	−6.94 (2.06)	0.55	−3.37	122	.001*	− 11.02; − 2.86
Psychosocial Health	15	70	76.29 (14.90)	99	82.41 (12.81)	−6.11 (2.14)	0.45	−2.86	167	.005	−10.34; − 1.89
Physical Functioning	8	70	82.00 (17.83)	99	91.41 (8.03)	−9.41 (2.28)	0.72	−4.13	89	<.001*	− 13.95; − 4.89
Emotional Functioning	5	70	71.57 (18.19)	99	79.24 (16.39)	−7.67 (2.67)	0.45	−2.86	167	.005	−12.96; −2.38
Social Functioning	5	70	81.86 (18.65)	99	86.67 (14.05)	−4.81 (2.64)	0.30	−1.82	122	.071*	− 10.03; 0.42
School Functioning	5	70	75.45 (19.39)	99	81.31 (16.28)	−5.86 (2.75)	0.33	−2.13	167	.035	−11.30; − 0.42
Parent Proxy-report											
Total Score	23	69	77.66 (13.99)	100	84.14 (11.00)	−6.48 (1.93)	0.53	−3.36	167	.001	−10.28; −2.67
Psychosocial Health	15	69	76.79 (14.52)	100	82.15 (11.79)	−5.36 (2.03)	0.41	−2.64	167	.009	−9.36; −1.34
Physical Functioning	8	69	80.26 (15.66)	100	90.11 (12.37)	−9.85 (2.25)	0.71	−4.37	123	<.001*	− 14.31; − 5.39
Emotional Functioning	5	69	70.91 (17.07)	100	78.40 (14.94)	−7.49 (2.48)	0.47	−3.02	167	.003	−12.39; − 2.60
Social Functioning	5	69	82.30 (18.88)	100	86.09 (14.52)	−3.79 (2.57)	0.23	−1.40	121	.163*	− 9.13; 1.55
School Functioning	5	69	77.17 (16.66)	100	81.95 (15.79)	−4.78 (2.53)	0.30	−1.89	167	.061	−9.77; 0.21

*N* number of participants, *SD* standard deviation, *df* degrees of freedom, *CI* confidence interval.

*Welch’s t used due to unequal variances, according to Levene’s Test for Equality of Variances

^a^Difference between patient and control sample mean scores

^b^Standard Error Difference

In parent proxy-reports, similar results were observed, with a 6.48 (SD = 1.93) score decrease in the Total Score (95% CI: -10.28 to − 2.67; *p* = .001) and a 9.85 (SD = 2.03) score decrease in the Psychosocial Health Summary Score in the patient sample (95% CI: -9.36 to − 1.34; *p* = .009). Physical Functioning domain was lower by 9.85 scores (SD = 2.25; 95% CI: -14.31 to − 5.39; *p* = <.001) and the Emotional Functioning domain by 7.49 scale scores (SD = 2.48; 95% CI: -12.39 to − 2.60; *p* = .001).

On further analysis we found no score differences between genders in patient and control groups. Comparing different age categories, age alone did not have an impact on the perceived HRQoL in most scores. However, hypertensive children aged 5-12 scored on average 18.00 (SD = 3.93) points lower in the Social Functioning Score, compared to hypertensive adolescents (95% CI: -25.87 to − 10.13; Hedges’ *g* = − 1.10; t(53) = − 4.59; *p* < .001). On the basis of this, we compared Social Functioning scores (using Welch’s *t*-test) between healthy and hypertensive children 5-12 years of age, and found a 12.52 (SD = 4.00) score decrease in hypertensive cases (95% CI: -20.42 to − 4.52; Hedges’ *g* = − 0.77; t(58) = − 3.13; *p* = .003). We can consequently say that although Social Functioning scores were not impaired in the hypertensive child and adolescent sample as a whole, hypertensive children experience a significant decrease in social performance.

Calculation of Pearson’s *r* correlation index revealed moderate to high correlations in child-parent pairs (see Table 2). In the patient sample, Pearson’s *r* values ranged from *r* = 0.62 to *r* = 0.79, and in the control sample from *r* = 0.56 to *r* = 0.80. Both Total Scores and Psychosocial Health Summary Scores had a high correlation in the patient (*r* = 0.79 and *r* = 0.75, respectively) and control samples (*r* = 0.79 and *r* = 0.80, respectively).

**Table 2:** Concordance of child self- and parent proxy-reports

	Child self-report	Parent proxy-report	
	Mean (SD)	Mean (SD)	Pearson’s r
Patient sample (*N* = 69)			
Total Score	77.85 (14.63)	77.66 (13.99)	0.79*
Psychosocial Health	76.51 (14.90)	76.79 (14.52)	0.75*
Physical Functioning	81.87 (17.93)	80.26 (15.66)	0.78*
Emotional Functioning	71.74 (18.27)	70.91 (17.07)	0.62
Social Functioning	81.88 (18.79)	82.30 (18.88)	0.78*
School Functioning	75.89 (19.18)	77.17 (16.66)	0.65
Control sample (*N* = 98)			
Total Score	84.90 (10.79)	84.44 (10.63)	0.79*
Psychosocial Health	82.69 (12.57)	82.38 (11.66)	0.80*
Physical Functioning	91.55 (7.95)	90.61 (11.11)	0.56
Emotional Functioning	79.69 (15.84)	78.62 (14.90)	0.75*
Social Functioning	86.79 (14.07)	86.21 (14.62)	0.71*
School Functioning	81.58 (16.14)	82.30 (15.50)	0.69

^*^Pearson’s *r* showing a strong correlation (> 0.7) between the patient and control sample, *p* < .001 for all values of Pearson’s *r*

*SD* standard deviation

## Discussion

In our study, we have compared the Health-Related Quality of Life of hypertensive children and adolescents from patients’ and parents’ perspectives to a control sample of primary school children and their parents, using a well-validated and frequently used PedsQL™ 4.0 Generic Core Scale Questionnaire [13, 15–18, 23]. Based on our results, both hypertensive children and adolescents as well as their parents as proxy-reports report lower overall HRQoL and Psychosocial Health, together with lower Physical and Emotional Functioning scores. Additionally, the patient sample had a significantly lower School Functioning score.

Calculation of Pearson’s *r* revealed moderate (*r* > 0.5) to high (*r* > 0.7) positive correlations between child self- and parent proxy-reports, ranges in accordance with [24]. This is higher than the publication on school children by Varni [23], with Pearson’s *r* values between 0.19 and 0.35, whereas some other studies have reported the correlation to be mostly in the moderate range; 0.69 in total scale scores in paediatric cancer patients [25] and from 0.36 to 0.64 in children with heart disease [26]. In contrast to experience in the literature [27], we did not find greater child-parent agreement in observable functioning (Physical HRQoL) in comparison to non-observable functioning (Psychosocial Health). Although the observed child-parent correlations in this study were higher than expected from literature, obtaining reports from both children and parents whenever possible is still advocated because of differences in correlations between studies.

Comparing the PedsQL™ 4.0 reports of our healthy control school-based sample to reports of Slovenia’s neighbouring countries [21, 28–30], displayed in Table 3, our self-report sample had the highest scores in all but the Social Functioning scale. The parent proxy-reports were second-highest in all scales, with Austrian parents reporting the highest scores in five out of six scales. Although the selected primary school enrols children from different socioeconomic and ethnical backgrounds, with such high overall scores we cannot rule out a potential selection bias as well as the effect arising from a low sample size. Alternatively, the high score results may be a reflection of overall good self-reported health of Slovenian children, as reported by OECD [31].

**Table 3:** Comparison of healthy population PedsQL™ scores with neighboring countries

Publication	Our Study	Felder-Puig et al. [20]	Berkes et al. [27]	Trapanotto et al. [28]	Jović et al. [29]
Region	Maribor, Slovenia	Vienna, Austria	Debrecen, Hungary	Padua, Italy	Zadar, Zagreb, Osijek and Split in Croatia
Sample Characteristics	99 Self- and 100 parent proxy-reports; ages 6 to 15 yr.; 46.5% boys, 53.5% girls	1412 children aged 8-12 yr.; 51.0% boys, 49.0% girls	366 Self- and 519 parent proxy-reports, ages 2-18 yr.; 55.5% boys, 44.5% girls	51 self- and 66 parent proxy-reports, aged 2-18 yr.; 21.2% boys, 78.8% girls	152 primary school children; 39.5% boys, 60.5% girls
	Mean (SD)	Mean (SD)	Mean (SD)	Mean (SD)	Mean (SD)
Patient sample					
Total Score	84.66 (11.00)	81.9 (12.6)	79.33 (12.35)	79.31 (10.00)	NA
Psychosocial Health	82.41 (12.81)	79.9 (14.1)	77.29 (13.39)	77.68 (10.56)	NA
Physical Functioning	91.41 (8.03)	87.8 (11.9)	83.12 (14.23)	82.54 (12.17)	77.85 (NA)
Emotional Functioning	79.24 (16.39)	76.9 (18.1)	72.10 (17.80)	72.16 (15.40)	68.08 (NA)
Social Functioning	86.67 (14.05)	82.6 (17.3)	83.81 (61.10)	80.98 (14.35)	90.47 (NA)
School Functioning	81.31 (16.28)	80.0 (16.7)	75.84 (16.65)	79.70 (12.58)	76.51 (NA)
Control sample					
Total Score	84.14 (11.00)	84.9 (10.5)	78.85 (13.18)	81.74 (11.21)	NA
Psychosocial Health	82.15 (11.79)	83.1 (11.6)	77.33 (13.69)	81.08 (11.63)	NA
Physical Functioning	90.11 (12.37)	90.6 (10.6)	81.03 (15.88)	82.99 (13.41)	NA
Emotional Functioning	78.4 (19.94)	79.4 (15.5)	71.79 (16.76)	75.53 (14.00)	NA
Social Functioning	86.09 (14.52)	88.1 (14.0)	84.45 (16.31)	83.79 (17.84)	NA
School Functioning	81.95 (15.79)	81.7 (14.2)	77.01 (16.93)	84.32 (13.67)	NA

*NA* Data not available, *yr.* years, *SD* standard deviation

On analysis of the size of mean differences between the patient and control groups, all the statistically significant differences also achieved the 5 points “cut-off” value for “minimally clinically significant difference”. Therefore, we can assume our results to be important in the clinical setting. In scores where no significant difference was observed, the mean score differences were below 5 score points.

Considering previously published literature, a meta-analysis of cross-sectional studies on HRQoL in the adult hypertensive population reported a slightly worse quality of life compared to normotensive individuals [11], results concordant with our own. However, in adolescents, findings from the German “KiGGS” study suggested higher self- and parent-rated quality of life, as also lower parent-rated emotional, conduct and overall problems with elevated blood pressure [10]. The authors explained this by absence of confounding physical comorbidity and the adolescents’ unawareness of being hypertensive. The latter partially explains the discrepancy with our findings, as we only recruited patients from the clinic, who were thus mostly aware of their disease. In comparison to the study by Wong et al. [12] on paediatric chronic kidney disease patients, which found no significant associations between elevated blood pressure and HRQoL, a lack of hypertensive disease symptoms or more dominant chronic kidney disease symptoms might have masked the HRQoL impairments of hypertension [12]. In our study, hypertension was the primary disease under consideration, thereby avoiding the risk of symptom misattribution. As a majority of hypertensive children report some symptoms of hypertension, including headache, insomnia, fatigue and chest or abdominal pain [32], impaired HRQoL seems a likely consequence.

When comparing the patient and control sample self-reports regarding to gender, no differences were found. However, in comparing age categories, we have found greatly reduced social functioning in hypertensive children compared to adolescents. While chronically ill children have been shown before to have impaired social functioning, although not diagnosis specific, age alone was not related with measures of social functioning [33]. Even though the frequent hospital visits might play a role in this difference, further research might be needed to confirm this finding.

### Limitations

Certain limitations regarding our study should be addressed. First, the standardized PedsQL Questionnaire did not contain questions regarding the duration or stage of the disease, treatment modality, nor gather information on socio-economic status or other social interactions at home or in school. However, in effort to preserve cross-study comparativeness of our results, no questions were added to the questionnaire that was standardized. The enrolled hypertensive participants were, however, from the same geographical region with similar socioeconomic backgrounds, in majority diagnosed with essential hypertension. In addition, they were mostly treated with lifestyle interventions, with a negligible number of cases necessitating drug therapy. Thus, we believe our participants have been similar in major characteristics. However, future studies on these topics influencing the quality of life with a high probability are needed.

Secondly, as it was a postal survey, we could not ensure that the parents followed the written instructions posted with the questionnaires. Especially important in this aspect is the high observed child-parent concordance, which might be the result of parents influencing their child’s responses. Second, the patient and control sample differed in gender, most patients being male and controls female. Furthermore, even though obesity has been previously shown to negatively influence HRQoL [34, 35], we did not consider it a confounding factor, as it plays a major role in the development of hypertensive disease.

Finally, findings in the control population showed exceptional HRQoL in both self- and proxy-reports in relation to neighbouring countries. While we believe this to be connected to well-organized and publicly available healthcare, educational and other government systems, our findings suggest additional studies are needed to explain these unique observations. It should also be stressed that children in our country have regular preventive clinical examinations performed every 3 years including blood pressure measurements, meaning that children of control group should have been truly healthy.

### Implications

The implications of our research are relevant in two aspects. First, we believe that the demonstrated study results give reason to assess the physical as well as psychosocial functioning of hypertensive children and adolescents, preferentially both from children’s as also parent’s perspectives. While the use of structured questionnaires measuring HRQoL in everyday paediatric clinical practice is under debate, according to Uzark et al. [36], utilizing such measures can facilitate patient-physician communication, improve patient/parent satisfaction, identify hidden morbidities, and assist in clinical decision-making. Moreover, it is advocated that specific interventions for deficits in HRQoL, including appropriate referrals, follow questionnaire findings, otherwise patient HRQoL outcomes might not be improved [8]. An example of such HRQoL-based interventions at a cardiology outpatient practice was presented by Uzark et al. [36]. Secondly, our findings indicate that hypertensive children differ from healthy controls in physical and psychosocial aspects. Therefore, special attention together with health-care fund allocation should be considered for this at-risk group of children, as early detection and intervention might prevent detrimental effects on psychosocial health and well-being later in life.

## Conclusion

This study demonstrates a significant and clinically relevant association of hypertension with lower physical and psychosocial functioning in a paediatric child and adolescent hypertensive sample. We observed a moderate to high correlation between child and parent proxy-reports. From reasons not yet fully understood, hypertensive children reported lower social functioning scores than hypertensive adolescents. Scores of healthy primary school children were slightly higher than scores of healthy children from neighbouring countries. Based on our findings, we suggest an evaluation of HRQoL in paediatric hypertensive patients and appropriate interventions based on the findings. Also, further research is needed to evaluate the influence of disease severity, duration, antihypertensive therapy and patient-related factors (e.g. illness-awareness) on the HRQoL outcome. Furthermore, insight into the resilience of hypertensive adolescents in the social functioning domain might give us further information on potential “protective” factors for psychosocial well-being of hypertensive children.

## Abbreviations

HRQoL: Health-related quality of life
PedsQL™ 4.0: Paediatric quality of life inventory version 4.0
